# Genetic variant in *SPAG16* is associated with the susceptibility of ACPA-positive rheumatoid arthritis possibly via regulation of *MMP-3*

**DOI:** 10.1186/s13018-022-03405-w

**Published:** 2022-11-24

**Authors:** Qingxi Lin, Bingxiang Zhou, Xiaoxiao Song, Wei Ye, Qinglong Li, Tong Shi, Chen Cheng, Yetian Li, Xing Wei

**Affiliations:** 1 Department of Orthopedics, Affiliated Taikang Xianlin Drum Tower Hospital, Medical School of Nanjing University, LinShan Road No. 188, Nanjing City, 210008 China; 2 Department of Orthopaedic Surgery, The JiangYan TCM Hospital of Taizhou City, JiangYan Road No. 699, Taizhou City, 225500 China; 3 Department of Orthopaedic Surgery, The First Affiliated Hospital of Anhui Medical University, Jixi Road No. 218, Hefei City, 230022 China

**Keywords:** Rheumatoid arthritis, Susceptible, *MMP-3*, ACPA-positive

## Abstract

**Objectives:**

In two previously published genome-wide association studies, a cluster of variants of *sperm-associated antigen16* (*SPAG16*) were reported to be associated with the radiological progression rate of ACPA-positive rheumatoid arthritis (RA) patients from North American and Southern European ancestry. In this study, we aimed to investigate whether the reported RA-risk loci in *SPAG16* are associated with the disease in the Chinese population and to further validate the functional role of the susceptible locus in RA tissues.

**Methods:**

A total of 500 ACPA-positive RA patients and 1000 age-matched healthy subjects were recruited. Two SNPs of *SPAG16*, including rs7607479 (C/T) and rs6435818 (A/C), were genotyped, and the genotyping data were compared with chi-square test. Gene expression analysis was performed in synovial tissues obtained from 40 RA patients and 30 non-RA controls surgically treated for bone fracture. The tissue expression of *SPAG16* and *matrix metalloproteinase 3* (*MMP-3*) was compared between the two groups by the Student’s t test. The relationship between serum indexes and mRNA expression of *SPAG16* and *MMP-3* were evaluated by Spearman’s correlation analysis.

**Result:**

For rs7607479, the frequency of genotype TT was significantly higher in RA patients than in the controls (49.0% vs. 40.4%, *p* = 0.002). The RA patients were found to have significantly lower frequency of allele C than the controls (30.9% vs. 36.8%, *p* = 0.001). As for rs6435818, there was no significant difference of genotype or allele frequency between the two groups. The mRNA expression of *MMP-3* was 1.63-fold higher in the RA patients than in the controls (*p* < 0.001). The expression of *SPAG16* was comparable between the two groups (*p* = 0.43). The mRNA expression of *MMP-3* was 1.39-fold higher in patients with genotype TT than in the patients with genotype CC (*p* = 0.006). The mRNA expression level of *MMP-3* was significantly correlated with serum rheumatoid factor (*r* = 0.498, *p* < 0.001) and C-reactive protein (*r* = 0.272, *p* = 0.01), weakly correlated with erythrocyte sedimentation rate (*r* = 0.236, *p* = 0.09).

**Conclusions:**

We validated a common genetic risk factor in ACPA-positive patients with RA, which is associated with the tissue production of *MMP-3* and disease progression. Further functional analysis into the role of rs7607479 in *MMP-3* expression can shed new light on the genetic architecture of ACPA-positive RA.

**Supplementary Information:**

The online version contains supplementary material available at 10.1186/s13018-022-03405-w.

## Introduction

Rheumatoid arthritis (RA) is a disease characterized by inflammatory changes of the synovial tissues [1, 2]. RA arises based on both genetic and epigenetic components including direct cigarette smoking, dust exposure and other environmental factors [3–5]. Previous studies have well documented the role of genetic predisposition in the development of RA [6–8]. As reported in earlier studies, the rate of RA for the general population is approximately 0.2–1% [9, 10]. For identical twins, the concordance of RA was reported to be 15% [11], which was much higher than that in the general population and thus indicated the importance of genetic factors for RA. In the past decades, a large number of genetic risk loci associated with RA has been discovered through genome-wide association studies (GWASs) in various populations worldwide [12–14]. The contribution of the genetic variation to RA was estimated to be about 60% [12–14].

For patients with RA, the presence of rheumatoid factor (RF), elevated C-reactive protein (CRP) level and high erythrocyte sedimentation rate (ESR) were reported to indicate the severity of disease [15]. In addition to RF, CRP and ESR, antibodies against citrullinated proteins (ACPA) have been also reported as a risk factor in the development of joint damage in RA patients [16, 17]. Positive detection of ACPA at early stage of RA can be prognostic of an increased likelihood of disease severity [16, 17]. As specific subgroups of RA patients, ACPA-positive individuals were found with higher risk of severe erosive phenotype and mortality rate than ACPA-negative individuals [18–20]. This variability of the clinical presentation between these two subgroups might reflect the presence of different underlying genetic risk factors. Analyzing ACPA-positive RA patients is therefore a useful strategy to reduce heterogeneity and increase the power to identify novel genetic factors associated with the severity of RA.

In a GWAS for ACPA-positive RA patients with radiographic joint damage, Knevel et al. [21] reported that a cluster of single nucleotide polymorphisms (SNPs), including rs7607479 (C/T) and rs6435818 (A/C), were associated with the radiological progression rate of RA in European and North American patients. Of note, rs7607479 reached genome-wide significance [21]. Interestingly, in a recently published case–control GWAS using ACPA-positive RA patients [22], rs6435818 was successfully located as a risk locus of RA in Caucasian and of Southern European ancestry. SNPs rs7607479 and rs6435818 were located in the intron of *sperm-associated antigen16* (*SPAG16*), which is predicted to have a role in the basal structure of the primary cilium [23]. To date, there was a lack of knowledge concerning the function of SPAG16. It is mainly expressed in sperm, testis and other tissues such as bone marrow [23]. Knevel et al. [21] confirmed that SPAG16 was also expressed in synovium tissues of RA, while there was a lack of relationship between SPAG16 expression and genotypes of rs7607479. Interestingly, Knevel et al. [21] observed that the genotype of rs7607479 was correlated with the serum level of a RA-associated gene, matrix metalloproteinase 3 (MMP-3). The MMPs are composed of a family of zinc- and calcium-dependent enzymes that contribute to the destruction of articular cartilage [24–27]. Abundantly expressed in the synovium tissues, MMP-3 is considered to be the main MMP involved in cartilage degradation of RA patients [28]. As reported in previous studies [29, 30], MMP-3 may be associated with the progression of the RA. Elevation of serum MMP-3 can be detected at both early stage and advanced stage of RA patients. Significant association between the promoter polymorphism of *MMP-3* and joint destruction was reported in patients with RA [31]. To our knowledge, there was a lack of replication of the association between *SPAG16* variants and the risk of RA in other populations. Moreover, the relationship between the genotype of *SPAG1*6 variants and the synovium mRNA expression of *SPAG16*/*MMP-3* was worthy of further investigation. In this study, we aimed to investigate whether the reported RA-risk loci rs7607479 and rs6435818 are associated with the disease in the Chinese population and to further validate the functional role of RA-risk loci.

## Methods

### Subjects

In this multicenter retrospective study, all RA patients fulfilling ACR/EULAR 2010 RA classification who came to our clinics between 2015 and 2021 were screened. Patients with other connective tissue diseases, including systemic lupus erythematosus, dermatomyositis or systemic sclerosis, were excluded from the study. ACPA was analyzed by a third-generation anti-cyclic citrullinated peptides (anti-CCP) enzyme-linked immunosorbent assay (ELISA) kit (DLD Diagnostika, Hamburg, Germany). Under the approval of the Ethics Committee of our institutions, a total of 500 ACPA-positive RA patients were recruited. The controls were composed of 1000 age-matched healthy subjects who underwent routine health examination organized by their employees. All the controls were excluded to have RA or other autoimmune diseases by clinical examinations. The baseline characteristics of the patients were recorded at their first visit, including age, gender, body mass index (BMI), ESR, CRP, and the serum level of RF.

### Blood collection

The ethylenediaminetetraacetic (EDTA)-anti-coagulated whole blood samples were collected from the patients and controls for DNA extraction. Genomic DNA was extracted from the leukocyte sediments by DNA extraction kit (QIAGEN, Tokyo, Japan) according to manufacturer’s guideline, which was then stored in − 20 °C for genotyping assays. Written informed consent forms were signed by each subject.

### Genotyping assays of rs7607479 and rs6435818

Two SNPs, including rs7607479 and rs6435818, were investigated in this study. Approximately 20 ng of the DNA sample was used for genotyping with TaqMan SNP Genotyping Assay. A total of 30 μl reaction volume, containing 4 μl of distilled deionized water, 4 μl of TaqMan Genotyping assay mix, 12 μl of genomic DNA and 10 μl of the TaqMan Genotyping master mix, was used for polymerase chain reaction (PCR) amplification. The results of genotyping assay were then analyzed on Roche LightCycler 480 II thermocycler (Roche Diagnostic Ltd., Basel, Switzerland). Ten percent of the samples were randomly selected to reproduce the genotyping results. Reproducibility of 100% was confirmed.

### Tissue expression of *SPAG16* and *MMP-3*

Synovial tissues were obtained from the knee joint of 40 RA patients with Parker-Pearson needle biopsy. Normal synovial tissues of 30 patients undergoing tibial plateau reconstruction due to traumatic fracture were used as the negative control. The total RNA was extracted from synovial tissues using TRIzol reagent (QIAGEN, Tokyo, Japan), which were then reversely transcribed with the PrimeScript RT Master Mix kit (TaKaRa, Tokyo, Japan). Tissue expression of *SPAG16* and *MMP-3* was quantified by Quantitative real-time PCR (qPCR) with beta-actin used as endogenous control. The specific primers were listed below, forward 5′- ATGTTCCAGATGTCTACACCCA -3′, reverse 5′- TGTAACTTCAACCCTTTGAGGTC -3′ for *SPAG16*, forward 5′- AGCAAGGACCTCGTTTTCATT -3′, reverse 5′- GTCAATCCCTGGAAAGTCTTCA -3′ for *MMP-3*, and forward 5′- CCTCGCCTTTGCCGATCC-3′, reverse 5′- GGATCTTCATGAGGTAGTCAGTC -3′ for beta-actin. All amplification reactions were carried out in triplicate.

### Statistical analysis

The SPSS software (version 22.0, Chicago, USA) was applied to statistical analysis. The Hardy–Weinberg equilibrium (HWE) test was performed to detect whether there could be selection bias of the subjects. The frequency of genotype and minor allele was calculated and compared between the cases and the controls by the chi-square test. The effect of 3 genetic models was examined, including the additive model, dominant model and recessive model. The odds ratio (OR) and 95% confidential intervals (CIs) were then calculated for the two SNPs. The tissue expression of *SPAG16* and *MMP-3* was compared between the two groups by the Student’s t test. One-way ANOVA was performed to compare the gene expression among different genotypes of rs7607479, and the Tukey test was applied to the post hoc pairwise analysis. The relationship between serum indexes and mRNA expression of *SPAG16* and *MMP-3* was evaluated by Spearman’s correlation analysis. Descriptive data were presented in the form of mean value ± standard deviation (SD). *p* value less than 0.05 was considered to indicate statistical significance.

## Results

### Baseline characteristics of the subjects

For association analysis, the patients and the controls were matched in terms of age (42.7 ± 11.9 yrs for cases vs. 43.3 ± 12.6 yrs for controls, *p* = 0.34), the ratio of male/female (144/356 for cases vs. 323/677 for controls, *p* = 0.18) and BMI (23.1 ± 4.3 kg/m^2^ for cases vs. 23.4 ± 5.7 kg/m^2^ for controls, *p* = 0.26). The mean value of RF was 92.1 ± 7.9 (IU)/ml. The mean ESR level and CRP level were 34.9 ± 10.3 mm/h and 58.5 ± 21.3 mg/L, respectively. The clinical characteristics of the subjects are presented in Table 1.

**Table 1:** Demographic data of the subjects

	RA patients (n = 500)	Non-RA controls (n = 1000)	*p*
Age (years)	42.7 ± 11.9	43.3 ± 12.6	0.34
Gender			
Male	144	323	0.18
Female	356	677	
BMI (kg/m^2^)	23.1 ± 4.3	23.4 ± 5.7	0.26
CRP (mg/L)	58.5 ± 21.3	N/A	N/A
RF (IU/ml)	92.1 ± 7.9	N/A	N/A
ESR (mm/h)	34.9 ± 10.3	N/A	N/A

### Association of rs7607479 and rs6435818 with RA risk

HWE test showed normal distribution of the genotype frequency of the subjects (*p* > 0.05). For rs7607479 of *SPAG16*, the distribution of genotype frequency of RA patients was significantly different from that of the controls (*p* = 0.005). As shown by the dominant model (Additional file 1: Supplementary table 1), the frequency of genotype TT was significantly different between the two groups (49.0% for cases vs. 40.4% for controls, *p* = 0.002). RA patients were found to have significantly lower frequency of allele C than the controls (30.9% for cases vs. 36.8% for controls, *p* = 0.001). The OR was 0.77 with 95% CI ranging from 0.65 to 0.90. As for rs6435818, there was no significant difference regarding genotype or allele frequency between the two groups (Table 2).

**Table 2:** Case–control comparison of the frequency of the genotype and allele for rs7607479 and rs6435818

rs7607479 (C/T)	Genotype			*p*	Allele		*p*	Odds ratio (95% CI^a^)
	CC	CT	TT		C	T		
Patients (n = 500)	54 (10.8%)	201 (40.2%)	245 (49.0%)	0.005	309 (30.9%)	691 (69.1%)	0.001	0.77 (0.65–0.90)
Controls (n = 1000)	140 (14.0%)	456 (45.6%)	404 (40.4%)		736 (36.8%)	1264 (63.2%)		

rs6435818 (A/C)	Genotype			*p*	Allele		*p*	Odds ratio (95% CI^a^)
	AA	AC	CC		A	C		
Patients (n = 500)	15 (3.0%)	153 (30.6%)	332 (66.4%)	0.41	183 (18.3%)	817 (81.7%)	0.35	0.91 (0.75–1.10)
Controls (n = 1000)	44 (4.4%)	308 (30.8%)	648 (64.8%)		396 (19.8%)	1604 (80.2%)		

### Expression of *SPAG16* and* MMP-3* in joint

**Fig. 1:**
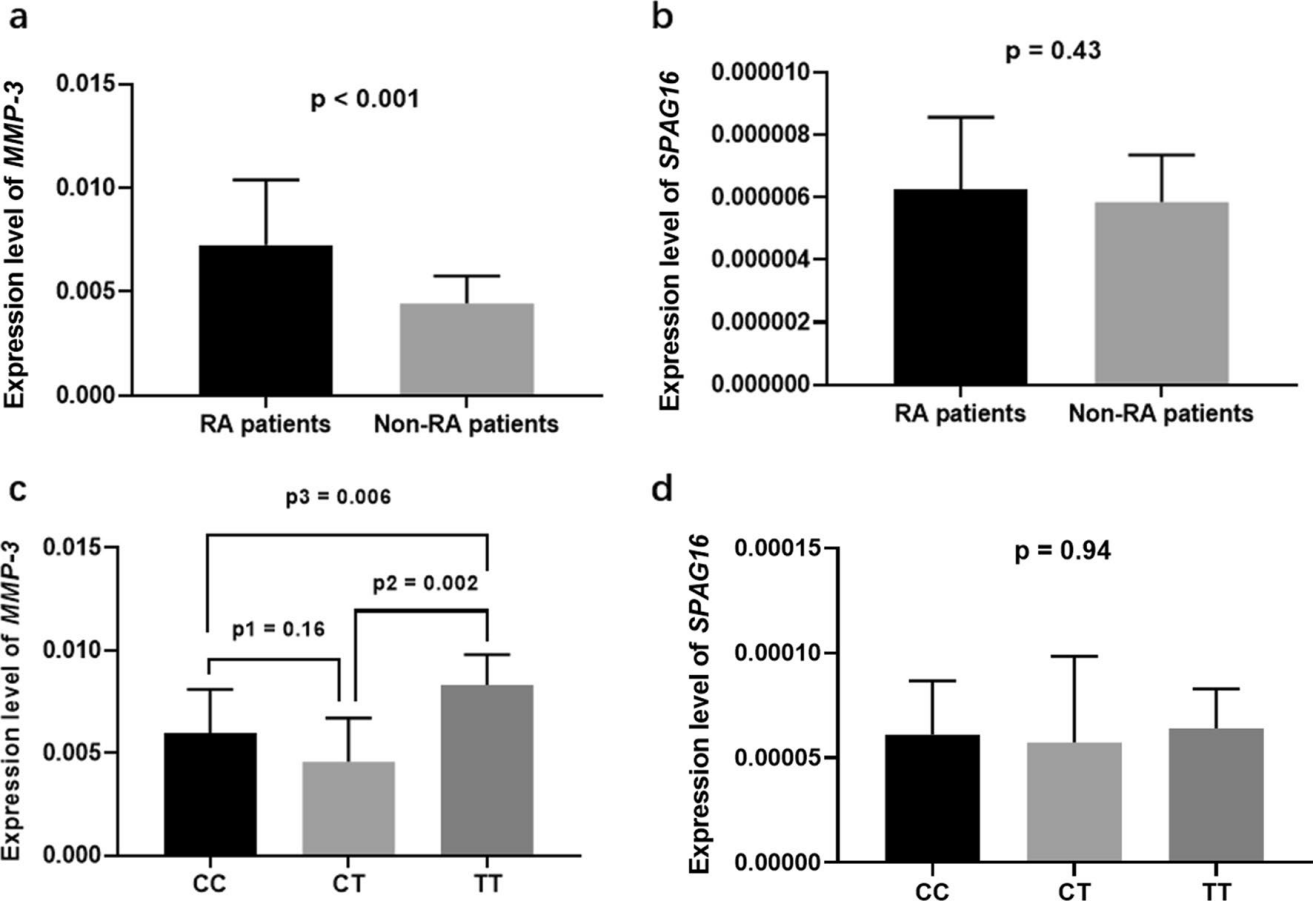
Tissue expression of *MMP-3* and *SPAG16* in RA patients. **a**, **b** Significantly higher expression of *MMP-3* was observed in RA tissues as compared with the non-RA controls. There was no significant difference regarding the tissue expression of *SPAG16* between the two groups;** c**, **d** There was significant difference regarding *MMP-3* expression among the three genotypes (*p* = 0.01). The mRNA expression of *MMP-3* was 1.39-fold higher in patients with genotype TT than in the patients with genotype CC (post hoc *p* = 0.006). There was significant difference regarding MMP-3 expression between genotype CT and genotype TT (post hoc *p* = 0.002). There was no significant difference between genotype CT and genotype CC (post hoc *p* = 0.16). There was no significant difference regarding the tissue expression of *SPAG16* among the three genotypes

For the 40 RA patients and 30 controls included in expression analysis, there was no significant difference in terms of age (42.3 ± 12.4 yrs for cases vs. 43.7 ± 11.2 yrs for controls, *p* = 0.62), the ratio of male/female (12/28 for cases vs. 14/16 for controls, *p* = 0.23), or BMI (23.7 ± 3.2 for cases vs. 24.1 ± 5.5 for controls, *p* = 0.70) between the two groups. As shown in Fig. 1a, the mRNA expression of *MMP-3* was 1.63-fold higher in the RA patients than in the controls (0.00725 ± 0.00314 for cases vs. 0.00443 ± 0.00132 for controls, *p* < 0.001). The expression of *SPAG16* was not statistically different between the two groups (0.0000625 ± 0.0000232 for cases vs. 0.0000585 ± 0.0000151 for controls, *p* = 0.43) (Fig. 1b).

For RA patients, there were 10 cases with genotype CC of rs7607479, 5 cases with genotype CT and 25 cases with genotype TT. There was significant difference regarding *MMP-3* expression among the three genotypes (*p* = 0.01). As shown in Fig. 1c, the mRNA expression of *MMP-3* was 1.39-fold higher in patients with genotype TT than in the patients with genotype CC (0.00833 ± 0.00148 for genotype TT vs. 0.00597 ± 0.00214 for genotype CC, post hoc *p* = 0.006). Also, there was significant difference between genotype CT and genotype TT (0.00457 ± 0.00241 for genotype CT vs. 0.00833 ± 0.00148 for genotype TT, post hoc *p* = 0.002). There was no significant difference between genotype CT and genotype CC (post hoc *p* = 0.16). As for the expression of *SPAG*16, there was no significant difference among the three genotypes (*p* = 0.95) (Fig. 1d). The tissue expression of *MMP-3* and *SPAG16* for different genotypes of rs7607479 is summarized in Additional file 1: Table S2.

**Fig. 2:**
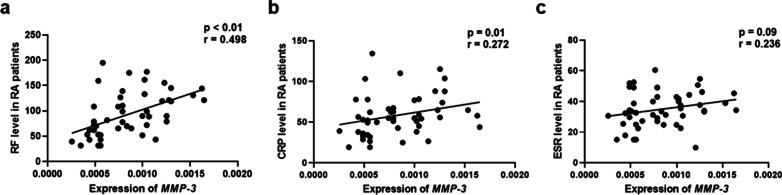
The relationship between *MMP-3* expression and clinical parameters of RA. The mRNA expression level of *MMP-3* was significantly correlated with the serum RF (*r* = 0.498, *p* < 0.001) and CRP (*r* = 0.272, *p* = 0.01). It was partially correlated with ESR (*r* = 0.236, *p* = 0.09)

As shown in Fig. 2, the mRNA expression level of *MMP-3* was significantly correlated with serum RF (*r* = 0.498, *p* < 0.001) and CRP (*r* = 0.272, *p* = 0.01), partially correlated with ESR (*r* = 0.236, *p* = 0.09). However, no significant correlation between *SPAG16* expression and these 3 clinical variables was found (*r* = 0.177 *p* = 0.21 for RF; *r* = 0.205, *p* = 0.15 for CRP; *r* = -0.166, *p* = 0.24 for ESR).

## Discussion

Previous GWASs have reported the association of different variants in *SPAG16* with the risk or severity of RA in Caucasian, North American and Southern European cohorts [21, 22]. To further validate the role of *SPAG16* in the susceptibility of RA, we replicated two previously reported variants and confirmed that rs7607479 of *SPAG16* was associated with RA in the Chinese population. We found that allele C of rs7607479 played a protective role in the risk of RA, which can significantly decrease the risk of RA by 0.77-fold. Consistent with our findings, Knevel et al. [21] reported a decreased RA risk of 0.71-fold in subjects with allele C from the North American population. As for the other reported susceptible locus of RA, rs6435818, we found no association with RA in the Chinese population. Julia et al. [22] discovered that rs6435818 was associated with RA in the European population through GWAS, although the combination of data in discovery stage and replication stages did not reach genome-wide significance. Herein, the lack of replication could be probably attributed to the ethnic difference or relatively small sample size of the current study. However, the association of rs6435818 with RA was worthy of further replication in other populations.

To determine the potential functional role of rs7607479 in RA, for the first time, we analyzed the tissue expression of rs7607479-related genes including *MMP-3 and SPAG16* in the synovial tissues of both RA patients and non-RA controls. Significantly higher tissue expression of *MMP-3* was observed in RA patients than in the controls. Moreover, patients with genotype TT of rs7607479 were found to have significantly increased expression of *MMP-3* than those with genotype CC. Interestingly, the expression of *SPAG16* was not statistically different between the patients and the controls. Moreover, there was no significant difference regarding the expression of *SPAG16* between patients with different genotypes of rs7607479. In line with our findings, Knevel et al. [21] observed that *SPAG16* expression did not correlate with rs7607479 genotypes in fibroblast-like synoviocytes, while patients with the allele C of rs7607479 had lower MMP-3 expression in serum levels. Taken together, it was possible that rs7607479 might be involved in the development of RA via regulation of *MMP-3*, although it was located in the intronic region of *SPAG16*. Interestingly, in this study, we confirmed that *MMP-3* is highly expressed in synovium tissues of ACPA-positive RA patients, and we observed significant correlation between *MMP-3* expression and serum level of RF and ESR. Collectively, increased *MMP-3* expression may indicate poor prognosis of the disease.

To date, the exact regulatory effect of rs7607479 on *MMP-3* expression remains obscure. Since we found no significant difference regarding the expression of *SPAG16* between patients and controls, it was not likely that the relationship between rs7607479 and *MMP-3* is mediated through interaction between *MMP-3* and *SPAG16.* Other unknown regulatory elements overlapped with the same genomic region of rs7607479 may play a role in this relationship. As speculated by Knevel et al. [21], miRNA encoded within the same intron of rs7607479 is worthy of further investigation to uncover the molecular mechanism underlying the regulation of *MMP-3* production by rs7607479.

The limitations of our study should be addressed here. First, we did not perform in vivo experiments to validate the role of rs7607479 in *MMP-3* expression. Further functional experiments are warranted to clarify the underlying regulatory mechanism. Second, the sample size of our study was smaller than previous GWAS study. Besides, no negative controls were included in the qPCR experiments, which could potentially lead to bias of the results. Although the power analysis confirmed that the sample size was sufficient, we believed that in future study larger sample size with negative controls may be included to further validate the relationship between *SPAG16* and RA. Third, it cannot be ruled out that the expression of *MMP-3* and *SPAG16* may be changed due to injury. In future study, tissues from normal controls need to be collected to better investigate if the gene expression of *MMP-3* and *SPAG16* was changed in RA patients.

## Conclusions

We validated a common genetic risk factor in ACPA-positive patients with RA, which is potentially associated with the tissue expression of *MMP-3* and disease progression. Further functional analysis into the role of rs7607479 in *MMP-3* expression can shed new light on the genetic architecture of ACPA-positive RA.

## Supplementary Information

**Additional file 1**.** Supplementary table 1**. Post-hoc analysis for the comparison of genotype frequency of rs7607479 between cases and controls.

## Data Availability

All data used in this study are available at the request of editors, reviewers and the research community.

## Abbreviations

RA: Rheumatoid arthritis
GWASs: Genome-wide association studies
BMI: Body mass index
RF: Rheumatoid factor
ESR: Erythrocyte sedimentation rate
CRP: C-reactive protein
HWE: Hardy–Weinberg equilibrium
OR: Odds ratio
Cis: Confidential intervals
ACPA: Antibodies against citrullinated proteins
MMP: Matrix metalloproteinases
