# Supplementary material for: Genetic variant in SPAG16 is associated with the susceptibility of ACPA-positive rheumatoid arthritis possibly via regulation of MMP-3
Source: J Orthop Surg Res. 2022 Nov 24;17:511. doi: 10.1186/s13018-022-03405-w (PMC9701044; doi:10.1186/s13018-022-03405-w)
Supplement: Supplementary file 1 — Additional file 1. Supplementary table 1. Post-hoc analysis for the comparison of genotype frequency of rs7607479 between cases and controls. [file 13018_2022_3405_MOESM1_ESM.docx]

**Supplementary table 1 Post-hoc analysis for the comparison of genotype frequency of rs7607479 between cases and controls**

|  | RA patients  (n = 500) | Controls  (n = 1000) | p |
| --- | --- | --- | --- |
| **Allele** |  |  | 0.001 |
| C | 309 (30.9%) | 736 (36.8%) |  |
| T | 691 (69.1%) | 1264 (63.2%) |  |
| **Additive model** |  |  | 0.005 |
| CC | 54 (10.8%) | 140 (14.0%) |  |
| CT | 201 (40.2%) | 456 (45.6%) |  |
| TT | 245 (49.0%) | 404 (40.4%) |  |
| **Dominant model** |  |  | 0.002 |
| CC+CT | 255 (51.0%) | 596 (59.6%) |  |
| TT | 245 (49.0%) | 404 (40.4%) |  |
| **Recessive model** |  |  | 0.08 |
| CC | 54 (10.8%) | 140 (14.0%) |  |
| TT+CT | 61 (89.2%) | 189 (86.0%) |  |

**Supplementary table 2 The mRNA expression of *MMP-3* and *SPAG16* for different genotypes of rs7607479**

| The mRNA expression | Genotype | | | p | Post-hoc testing | | |
| --- | --- | --- | --- | --- | --- | --- | --- |
|  | CC  (n = 10) | CT  (n = 5) | TT  (n = 25) |  | p1 | p2 | p3 |
| ***MMP-3*** | 0.00597 ± 0.00214 | 0.00457 ± 0.00241 | 0.00833 ± 0.00148 | 0.01 | 0.16 | 0.002 | 0.006 |
| ***SPAG16*** | 0.0000611 ± 0.0000256 | 0.0000573 ± 0.0000412 | 0.0000641 ± 0.0000187 | 0.94 | 0.82 | 0.76 | 0.64 |

The ANOVA test was used for statistical analysis. The Tukey test was applied to the post hoc pairwise analysis. p1 indicated subgroup comparison between CC and CT, p2 indicated subgroup comparison between CT and TT, and p3 indicated subgroup comparison between CC and TT.
